# Current Production Capability of Drug-Resistant Pathogen Enables Its Rapid Label-Free Detection Applicable to Wastewater-Based Epidemiology

**DOI:** 10.3390/microorganisms10020472

**Published:** 2022-02-20

**Authors:** Waheed Miran, Xizi Long, Wenyuan Huang, Akihiro Okamoto

**Affiliations:** 1 School of Chemical and Materials Engineering, National University of Sciences and Technology, Islamabad 44000, Pakistan; waheed.miran@scme.nust.edu.pk; 2 International Center for Materials Nanoarchitectonics (WPI-MANA), National Institute for Materials Science, 1-1 Namiki, Tsukuba 305-0044, Japan; long.xizi@nims.go.jp (X.L.); huang.wenyuan@nims.go.jp (W.H.); 3 Graduate School of Chemical Sciences and Engineering, Hokkaido University, North 13 West 8, Kita-ku, Sapporo 060-8628, Japan

**Keywords:** antibiotic-resistant pathogen, wastewater-based epidemiology, whole-cell electrochemistry, single-potential amperometry, extracellular electron transport

## Abstract

A rapid and label-free method for the detection of drug-resistant pathogens is in high demand for wastewater-based epidemiology. As recently shown, the extent of electrical current production (*I_c_*) is a useful indicator of a pathogen’s metabolic activity. Therefore, if drug-resistant bacteria have extracellular electron transport (EET) capability, a simple electric sensor may be able to detect not only the growth as a conventional plating technique but also metabolic activity specific for drug-resistant bacteria in the presence of antibiotics. Here, one of the multidrug-resistant pathogens in wastewater, *Klebsiella pneumoniae*, was shown to generate *I_c_*, and the extent of *I_c_* was unaffected by the microbial growth inhibitor, kanamycin, while the current was markedly decreased in environmental EET bacteria *Shewanella oneidensis*. Kanamycin differentiated *I_c_* in *K. pneumonia* and *S. oneidensis* within 3 h. Furthermore, the detection of *K. pneumoniae* was successful in the presence of *S. oneidensis* in the electrochemical cell. These results clarify the advantage of detecting drug-resistant bacteria using whole-cell electrochemistry as a simple and rapid method to detect on-site drug-resistant pathogens in wastewater, compared with conventional colony counting, which takes a few days.

## 1. Introduction

Because of the extensive use of antibiotics for treating human and animal infectious ailments, an enormous amount of antibiotics of therapeutic origin are found in various anthropic environments, such as sewage and wastewater treatment plants [1,2]. The widespread use of antibiotics has led to the spread of antibiotic-resistant pathogens, which currently represent a major public health concern [3,4]. Therefore, the risk of drug-resistant bacterial pathogens in wastewater compels the monitoring of wastewater for different types of microbial pathogens. For an appropriate risk assessment, an effective tool that is inexpensive, rapid, label-free, easy to perform, and can be run in high numbers for the detection of drug-resistant pathogens in wastewater bodies is in great demand [5]. Wastewater-based epidemiology can be a very useful methodology for government agencies for estimating the prevalence of antibiotic-resistant pathogens, especially in developing countries where such information is scarce due to financial limitations and the lack of well-structured investigations and surveillance [6]. Current techniques, such as agar plating, and polymerase chain reaction, used for the detection of different types of drug-resistant microbial pathogens contaminating water and wastewater, are time-consuming and expensive [7,8].

Recently, electrical current production via extracellular electron transport (EET) has been shown to indicate metabolic activity [9,10]. Monitoring the generation of electrical current as an indicator of cellular metabolic activity in pathogens represents a new direction for research aimed at assessing and screening the effects of antimicrobials on pathogenic metabolic activity [11]. Therefore, if drug-resistant bacteria have EET capability, the simple electric sensor may be able to detect not only the growth but also the metabolic activity specific for drug-resistant bacteria in the presence of antibiotics and can be a new tool for pathogen detection without gene engineering and labeling [12].

In the present work, we conducted a whole-cell electrochemical assay using *Klebsiella pneumoniae*, a pathogen frequently found in wastewater streams, and *Shewanella oneidensis* MR1, a model environmental bacterium. We selected a multi-drug-resistant *K. pneumoniae*, which is a common hospital-acquired pathogen that causes contagions with large morbidity and mortality rates as high as 50% [13]. Furthermore, the *K. pneumoniae* resistome evolves under antibiotic and biocide selective pressures, resulting in extremely drug-resistant or high-risk clones, with huge epidemic potential [14]. For control experiments, a non-drug-resistant environmental EET-capable model strain *Shewanella oneidensis* MR1 was selected, and the impact of the drug on the current production of both bacteria was determined.

## 2. Materials and Methods

### 2.1. Cell Culture Preparation

*KlebsiellaPneumonia* KPNIH1, obtained from Manoil Lab (Washington University, Seattle, WA, USA), was precultivated in 30 mL Luria-Bertani (LB) broth medium, in 50 mL falcon tubes at 37 °C. The cells were grown until late exponential growth phase followed by centrifugation at 7800 rpm for 10 min, and the resultant cell pellet was washed with minimal defined medium (DM) twice. DM was composed of 2.5 g/L NaHCO_3_, 0.08 g/L CaCl_2_·2H_2_O, 1.0 g/L NH_4_Cl, 0.2 g/L MgCl_2_·6H_2_O, 10.0 g/L NaCl, 7.2 g/L HEPES.

*S. oneidensis* MR-1 was cultured with 10 mL LB medium at 30 °C for 20 h in aerobic conditions by picking a single colony from LB solid medium plate. Then the bacteria were washed twice using DM. The cells were centrifuged at 7800× *g* for 10 min, followed by supernatant removal. Cell pellets obtained after centrifugation were resuspended in 10 mL DM. To remove the reductive energy, cells were pre-cultured in the DM for 4 h within the anaerobic condition and then washed by DM medium. Finally, cell OD was adjusted to the required values by DM as the final concentration in electrochemical cells.

### 2.2. Whole-Cell Electrochemical Analysis

Electrochemical measurements were conducted in a single-chamber, three-electrode system consisting of an indium tin-doped oxide (ITO) glass substrate (surface area of 3.1 cm^2^) as the working electrode at the bottom of the glass chamber, and Ag/AgCl (KCl saturated) and a platinum wire, which were used as reference and counter electrodes, respectively [15,16]. We used 5 mL DM containing 10 mM glucose as an electrolyte, and the solution was purged with N_2_ gas for at least 15 min to remove the dissolved oxygen in the electrochemical cell. The cell collected from the growth medium after centrifugations were adjusted to the required OD_600_ (0.001 to 0.5) by diluting with DM. Potentiostatic condition of +0.4 V vs. a standard hydrogen electrode (SHE) was maintained for single potential amperometry (SA) measurements. During the electrochemical measurements, the reactor temperature was maintained at 37 °C and operated without agitation. The potentiostat (VMP3, BioLogic Company, Seyssinet-Pariset, France) was used to perform the techniques and measurements, such as SA and differential pulse voltammetry (DPV), as previously described [16,17]. DPV was conducted under the following conditions: 5.0 mV pulse increments, 50 mV pulse amplitude, 300 ms pulse width, and a 5.0 s pulse period.

### 2.3. Scanning Electron Microscopy

At the end of the electrochemical experiment, supernatant was removed, and the ITO electrodes were detached from the reactor. Cells were fixed with 2.5% glutaraldehyde and then washed twice with phosphate buffer solution (PBS). The dehydration of washed samples was carried out with 25, 50, 75, 90, and 100% ethanol gradients in the PBS for 10 min each. Further, samples were dried three times with t-butanol and then freeze-dried under a vacuum system. The samples obtained after freeze-drying were subject to coating with evaporated platinum and subsequently visualized by Keyence VE-9800 microscope.

### 2.4. Crystal Violet Staining

ITO electrodes from the reactor (each reactor had one ITO electrode) before and after the current production experiment were washed three times with PBS to remove the planktonic cells. For the electrode before the current production, we stored the electrode in a cell suspension for 36 h at 4 °C to suppress the growth and terminate spontaneous irreversible adhesion to the electrode surface [18]. Cells attached to the ITO surface were stained using 0.1% crystal violet for 10 min, followed by washing three times with PBS to remove unbound crystal violet. A mixture of acetone and ethanol (3:1) was used to release bound crystal violet. The absorbance value was measured at 595 nm using an absorption plate reader.

### 2.5. Antibiotic Solution

A stock solution of kanamycin sulfate 50 mg/mL (dissolved in demineralized water) was purchased from FUJIFILM Wako Pure Chemical Corporation, Osaka, Japan. The stock solution was added to the electrochemical reactor containing bacterial cells and DM to a final concentration of 2 mg/mL. This concentration of kanamycin is considered a lethal concentration for non-drug-resistant pathogens. The antibiotic solution was mixed well in the reactor and kept for at least 30 min before the start of electrochemical measurements.

### 2.6. Live Dead Assay

BacLight LIVE/DEAD Bacterial Viability Kit (ThermoFisher Scientific, Shibaura, Japan) was used in our experiments to assess live dead bacteria. The kit contains two nucleic dyes, SYTO 9 and propidium iodide (PI). SYTO 9 is a membrane permeant, while PI is a membrane impermeant dye. Saline medium (0.9% NaCl) was used for diluting the stock dyes 100 times to make working solutions of SYTO 9 and PI. Prior to fluorescence microscopy, 30 µL of the working solution was added to the ITO-attached cells (obtained after the electrochemical experiment) after gentle washing with saline medium. Samples were placed in the dark for 15 min at room temperature and washed gently with saline medium to remove the excess dyes. Samples were visualized with a Leica DFC45 C epifluorescent microscope.

## 3. Results and Discussion

### 3.1. K. pneumoniae Produces Electric Current via Glucose Oxidation

**Figure 1:**
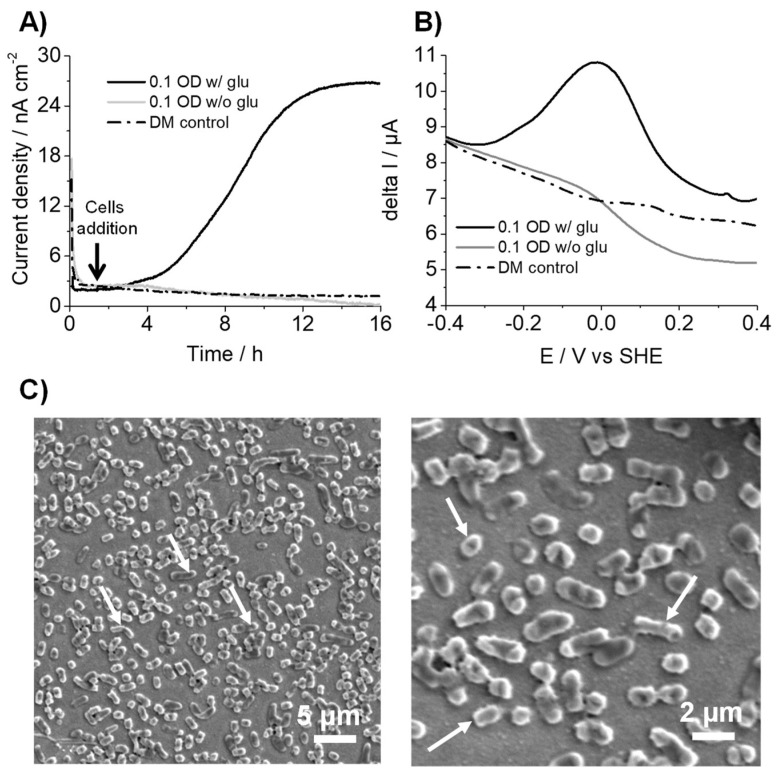
(**A**) Time course for the current production of *K. pneumoniae* in the presence of 10 mM glucose on an ITO electrode at +0.4 V (vs. SHE). (**B**) Differential pulse voltammograms for *K. pneumoniae* on an ITO electrode surface with and without glucose addition at the end of the current measurement experiment. (**C**) Scanning electron microscopy images of *K. pneumoniae* cells attached to the electrode surface showing rod-shaped morphology. Arrow indicates cells. Experiments were repeated at least twice, and representative data is presented.

We first explored the current production capability of *K. pneumoniae* in a three-electrode electrochemical reactor system using SA. When 10 mM glucose as the sole electron donor was used, *K. pneumoniae* generated an anodic current at +0.4 V (vs. SHE) and an initial 0.1 OD_600_ (Figure 1A). Upon the addition of cells, the current gradually increased to approximately 25 nA cm^2^ in 12 h, suggesting electron transport. In contrast, there was no current generation without glucose or *K. pneumoniae*, which is indicative of the current production association with glucose oxidation by *K. pneumoniae* cells (Figure 1A). DPV measurements were performed to characterize the energy levels of the potential electron transfer pathways between cells and electrodes. DPV measurements showed a clear redox peak at approximately +10 mV (vs. SHE) with *K. pneumoniae* in the reactor (Figure 1B). In contrast, no significant peak was observed without cells (Figure 1B), suggesting that *K. pneumoniae* contained redox agents that potentially mediated electron transport to the electrode surface. The redox potential observed was close to that reported for cytochromes. In addition, half-width potential was broader for outer membrane cytochrome that mediates direct EET mechanisms to electrode surfaces, as in *S. oneidensis* [16,17], suggesting that *K. pneumoniae* may also be capable of direct electron transfer. However, indirect electron transfer via redox mediators cannot be ruled out; since no further analyses were performed, either a mediator or a protein could be responsible for the redox peak observed. Accordingly, scanning electron microscopy of the ITO electrode surface after current production revealed that cells were attached to the electrode surface (Figure 1C), signifying cell attachment to the electrode surface associated with the oxidative signal in the differential pulse voltammogram. These results strongly suggested that *K. pneumoniae* has a direct EET capacity associated with glucose oxidation metabolism.

### 3.2. K. pneumoniae Grows on the ITO Electrode Surface

**Figure 2:**
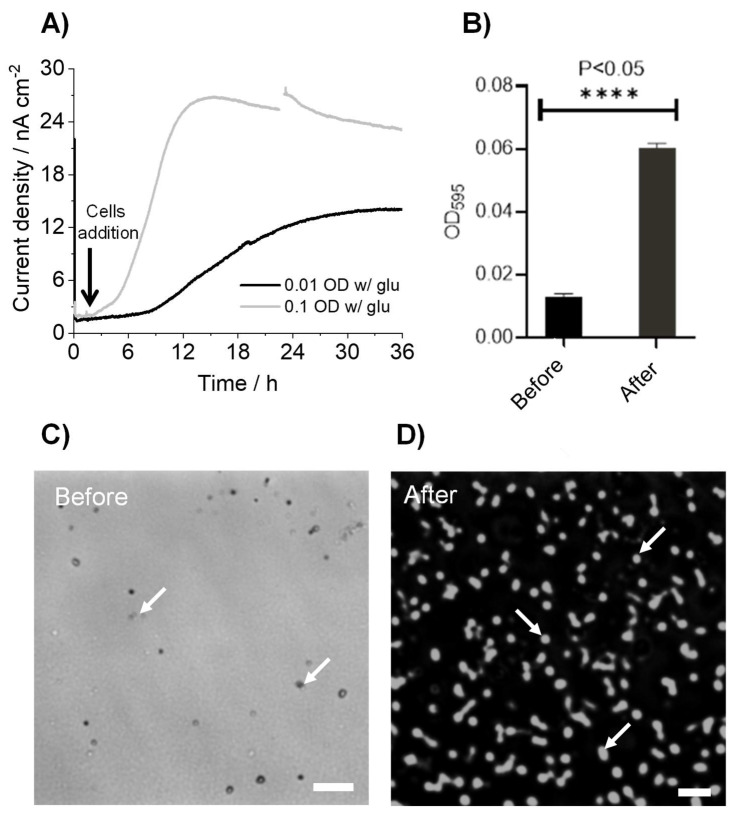
(**A**) Time course for the current production of *K. pneumoniae* in the presence of 10 mM glucose on an ITO electrode at +0.4 V (vs. SHE) initiated with OD_600_ of 0.1 and 0.01. (**B**) Quantification of *K. pneumoniae* cell numbers using crystal violet staining on the ITO electrode surface before and after current production. One-way analysis of variance with Tukey-Kramer comparison test was performed (**** *p* < 0.05). (**C**,**D**) Microscopy images of *K. pneumoniae* cells before and after current production on the ITO electrode surface. Scale bar, 10 µm. Arrow indicates cells.

Next, we examined whether the observed current production could associate with the growth of *K. pneumoniae* cells on the ITO electrode surfacewhen electrode incubation initiate at low cell density. The electrochemical measurements at +0.4 V (vs. SHE) and a lower 600 nm optical density (OD_600_) of 0.01 showed that the current production rise was delayed compared with those at an OD_600_ of 0.1 (Figure 2A). Maximum current production was only halved, suggesting that the time before the increase and production of the current were limited by the cell density on the ITO electrode. To examine cellular attachment on the ITO electrode, relative quantification of the number of cells attached to the ITO electrode surface was performed using a crystal violet staining assay before and after the current measurement for 36 h. Figure 2B clearly shows that the cell number increased by six folds on the ITO electrode during the current production, compared to a control electrode before current production. Accordingly, microscopy confirmed a substantial difference in the number of cells on the electrodes under the same conditions (Figure 2C,D). These results suggested that the current production by *K. pneumoniae* and its increase are assignable to metabolic activity and cell growth, respectively, in the electrochemical reactor, which can be used to assess the impact of antibiotic agents.

### 3.3. Detection of Drug-Resistant Pathogens Using Current Producing Capability

**Figure 3:**
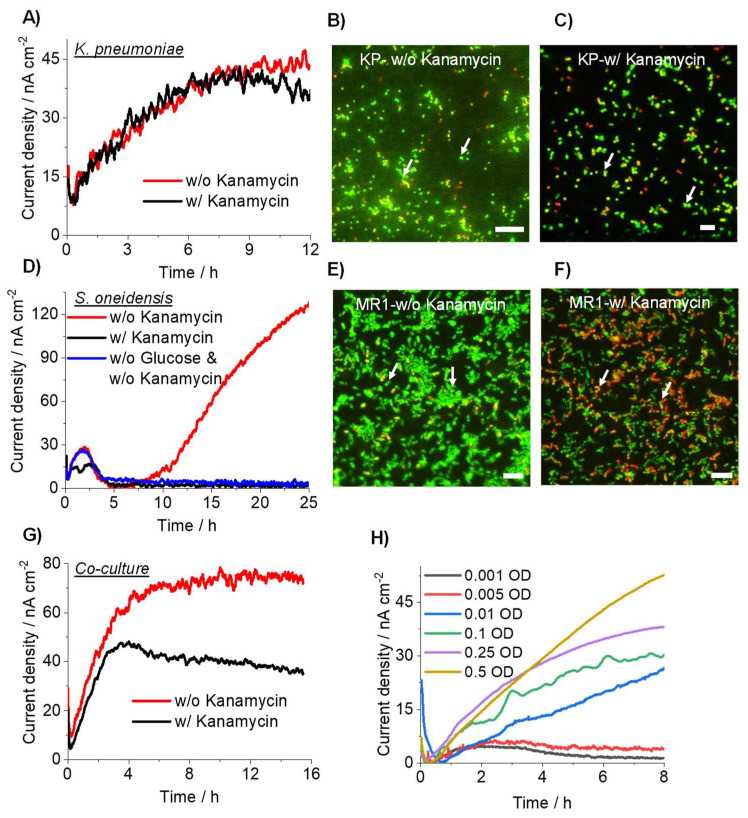
Time course for current production of *K. pneumoniae* (**A**) and *S. oneidensis* (**D**) in the presence of 10 mM glucose on an ITO electrode at +0.4 V (vs. SHE) and an OD_600_ of 0.1 with and without kanamycin. A mixed population of live and dead *K. pneumoniae* (**B**,**C**) and *S. oneidensis* cells (**E**,**F**) stained using the Fluorescence Live/Dead Bacterial Imaging Kit. Live bacteria expressed green fluorescence, while dead cells with damaged membranes expressed red fluorescence. (**B**,**E**) is without kanamycin, while (**C**,**F**) is with kanamycin. Scale bar, 10 µm. Arrows indicate cells. (**G**) Time course for current production in the coculture, *K. pneumoniae* (0.1 OD) and *S. oneidensis* (0.01 OD) in the presence of 10 mM glucose on an ITO electrode at +0.4 V (vs. SHE) with and without kanamycin. (**H**) Time course for current production from *K. pneumoniae* in the presence of 10 mM glucose on an ITO electrode at +0.4 V (vs. SHE) using an OD_600_ of 0.001 to 0.5 with kanamycin.

To examine whether drug resistivity is detectable with the microbial current production associated with metabolic activity, current production was compared between kanamycin-resistant *K. pneumoniae* and *S. oneidensis* in the presence of kanamycin. *K. pneumoniae* in the presence of 10 mM glucose as the carbon source and an OD_600_ of 0.1 showed no significant difference with or without kanamycin addition at 2 mg/mL (Figure 3A), consistent with kanamycin resistance in *K. pneumonia*. The live-dead assay also showed no substantial difference between the presence and absence of kanamycin (Figure 3B,C). In contrast, the presence of kanamycin at the same concentration suppressed the current production of the non-kanamycin-resistant bacterium *S. oneidensis*, compared with that in the absence of kanamycin (Figure 3D). The current production was observed in the first 2 h in the absence of glucose, suggesting that the initial current production was attributable to the reductive energy from the pre-culture conditions. The live-dead assay confirmed the presence of a large number of dead cells in the presence of kanamycin (Figure 3E,F). Furthermore, the electrochemical detection of *K. pneumoniae* was also successful in the coculture with *S. oneidensis*. The current production decreased in the coculture in the presence of kanamycin, and the extent of the decrease was consistent with the current production in a pure culture of *S. oneidensis* (Figure 3G). While the increase in current was not observed, the current production was continuously observed for 16 h, suggesting that the growth of *K. pneumoniae* was not clear in the presence of *S. oneidensis*. However, its metabolic activity was sustained. These results show that microbial current production in antibiotics reflects only the metabolic activity of drug-resistant bacteria.

Finally, we examined the detection limit of *K. pneumoniae* in the presence of kanamycin. Upon varying the initial cell concentration, the increase in current production was detectable until an OD_600_ of 0.01, but not <0.005, which was reached in the presence of an antibiotic agent (Figure 3H). This suggests that kanamycin impacts the electron transfer to electrode by *K. pneumonia* at very low cell concentrations. Given that *S. oneidensis* current production was observed in the first 2 h, current production after 3 h represents the metabolic activity of drug-resistant bacteria, which is considerably shorter than conventional plating methods that require 24 to 72 h [19]. Therefore, the lowest cell density for differentiating *K. pneumonia* from *S. oneidensis* was at an OD_600_ of 0.01. For example, if the bacteria from wastewater were concentrated to OD_600_ 1.0, 1% *K. pneumoniae* abundance would be detectable, which is comparable with plating technology [20]. Furthermore, because we used minimum medium DM as an electrolyte to avoid contamination, a richer medium may help to improve the minimum detection limit.

## 4. Conclusions

Our proof-of-concept study based on bioelectrochemical sensors showed that the EET-capable drug-resistant pathogens could be detected by utilizing their current generation ability, which is related to their metabolic activity and not affected by the presence of an inhibitor. The preliminary data from this work shows that the current production capacity of drug-resistant pathogens can help in assessing their presence in a mixture of bacteria by utilizing their cellular metabolism. Compared with plating techniques, current production rapidly detects the presence of drug-resistant bacteria and discriminates between bactericidal and bacteriostatic effects. Furthermore, plate counting underestimates the presence of bacteria as quiescent and viable but not culturable, and nonculturable microorganisms are omitted from the count [21]. Based on our technique, epidemiological data from the field can be effectively used by government agencies for emergency readiness and actions, forecasting disease spread in a population, and building statistical and mechanistic disease models for effective emergency handling [22]. Sensitivity can be overcome by the enhancement of current production with the use of suitable mediators, richly defined medium, or modified electrodes.
